# Association between Dietary Diversity and Weight Status of Aboriginal Primary School Children in Negeri Sembilan, Malaysia

**DOI:** 10.21315/mjms2022.29.1.10

**Published:** 2022-02-23

**Authors:** Divanirsh Devindran, Vaidehi Ulaganathan, Zhe Yee Oeh, Lih Xuan Tan, Silambarasi Kuralneethi, Zen Yang Eddie Eng, Lih Shiow Lim, Wei Ni Grace Chieng, Jia Ling Tay, Sook Yee Lim

**Affiliations:** Faculty of Applied Sciences, UCSI University, Kuala Lumpur, Malaysia

**Keywords:** aborigine children, food groups, diet diversity status, weight status

## Abstract

**Background:**

The purpose of this study is to determine the association between dietary diversity and weight status of aboriginal primary school children.

**Methods:**

Dietary diversity measures food intake diversity in food groups, whereas weight status indicates nutritional status. Dietary serving score (DSS) method was used to determine dietary diversity status, while weight status was assessed using BMI-for-Age (BAZ).

**Results:**

Results reported that 51.9% and 54.2% were male and 10 years old-12 years old children, respectively. A total of 36.4% of children consumed cereal/grains diversely while fruits were not diversely consumed by 96.4% of them. Approximately 60.8% of children were reported to have normal weight status, which was followed by overweight (17.7%), obese (16.7%) and thinness to severe-thinness (4.8%). There was an association between DSS of fruits, meat/fish/ eggs, legumes/lentils and milk/dairy products with age group, vegetables DSS with gender and BAZ with parental employment status (*P* < 0.05). The association between DSS of all food groups and total with BAZ were reported to be insignificant, indicating no association between both variables (0.00 < *r* < 0.30; *P* > 0.05).

**Conclusion:**

Children from this study were shown to practice a monotonous diet, although the majority of them were within normal weight status.

## Introduction

Malnutrition refers to the excess, insufficient or imbalanced nutrient intake from a person’s diet (1). Malnutrition has an adverse impact on children’s growth and development in which the severity is determined based on the degree of malnutrition. Some of these impacts include stunting (low Height-for-Age), underweight (low Weight-for-Age), wasting (low Weight-for-Height), as well as overweight and obesity (high Weight-for-Age). Malnourished children are susceptible to deprived cognitive development apart from the inability to achieve optimal growth and development. It was discovered that the proportion of underweight among children with learning disabilities was 3.4%–3.65%, while 7.6%–37% and 5.7%–52% were reported as overweight and obese, respectively (2). Moreover, they are at higher risk of mortality, whereby childhood mortality among children aged below 5 years old is approximately 45% in low and middle-income countries (1).

Weight status is an effective indicator of malnutrition. Weight status is categorised as severe thinness, thinness, normal, overweight and obese (1). The cause of under-nourishment (severe thinness and thinness) is due to insufficient energy intake to meet the requirement, whereas over-nourishment (overweight and obese) is the chronic mismatch of energy intake and expenditure, which gives rise to excess energy accumulation (3). Among Malaysian children aged 5 years old–14 years old, the prevalence of obesity rose by 8.1%, while the prevalence of thinness decreased by 4.4% (5 years old–9 years old) and 1.8% (10 years old–14 years old) between 2011 and 2015 (National Health and Morbidity Survey III [NHMS III]) (4). Environmental factors such as diet quality could influence the weight status of children (5).

Diet diversity is a qualitative measure of food intake respective to food groups, reflecting the availability and accessibility of households to various food items of different food groups (6). To achieve optimal growth and development, food items from different food groups, namely cereals/grains, fruits, vegetables, legumes/lentils, meat/fish/eggs and milk/ dairy products, should be consumed within the energy requirement (7). Dietary diversity score (DDS), DDS-half serving (SDDS), food variety score (FVS) and dietary serving score (DSS) are examples of tools used to assess one’s diet diversity status respective to food groups (8). The purpose of this study is to determine the association between dietary diversity and weight status of aboriginal primary school children.

## Methods

### Study Design and Participants

This is an analytical cross-sectional study. It was conducted in three aboriginal primary schools in Negeri Sembilan, Malaysia, which are Sekolah Kebangsaan Sungai Sampo, Bandar Seri Jempol; Sekolah Kebangsaan Tekir, Labu and Sekolah Kebangsaan Putra, Simpang Durian. The participants of this study were aborigine children aged from 7 years old–12 years old and their respective parents. The inclusion criteria were parents who can read and converse in Bahasa Malaysia or English and aborigine children of all religions and beliefs. Krejcie and Morgan’s (9) table was referred to determine the sample size which was approximately 210 aborigine children based on the population of children in three schools. The 210 study samples were chosen using the convenience sampling method.

### Study Instruments

An interviewer-administered questionnaire was used to record the dietary intake and socio-demographic characteristics of study samples. Furthermore, the Tanita^®^ digital weighing scale and Seca mobile stadiometer were used to record anthropometric measurements.

### Serving Size

Serving Size (SS) was used to quantify the amount of food intake by food groups (cereals/ grains, fruits, vegetables, legumes/lentils, meat/ fish/eggs and milk/dairy products) by frequency of intake (daily, weekly or yearly).

### Dietary Serving Score

The dietary serving score (DSS) method was used to determine diet diversity status. DSS methods determine the diet diversity status of food groups based on the number of servings, the number of food items of each food group and age group (Table 1).

### BMI-for-Age

WHO BMI-for-Age (BAZ) charts for 5 years old–9 years old were used to determine the weight status of aborigine children.

### Data Analysis

Data analysis was conducted using the WHO Anthroplus software and Statistical Package for the Social Sciences (SPSS) version 25.0 software. Weight status was determined using WHO Anthroplus software. Moreover, SPPS software version 25.0 was used to determine the means, percentages, frequencies and standard deviations of collected data. One-way ANOVA and independent *t*-tests were used to determine the mean differences between independent and dependent variables with confounding variables of this study. Furthermore, Spearman correlation and partial correlation tests adjusted for age, gender, and parental employment status were conducted to determine the association between continuous independent (i.e. diet diversity) and dependent (i.e. weight status).

## Results

### Descriptive Analysis of Study Variables

Based on our results, cereal/grains serving score (SS) and vegetables SS recorded interquartile range (IQR) values of 5.25 and 2.18, respectively, while fruits SS and legumes/lentils SS have recorded 9.54 and 6.41, respectively. In addition, meat/fish/eggs SS have recorded an IQR value of 18.71, whereas milk/dairy products SS have recorded a value of 1.36. Cereal/grains DSS reported an IQR value of 0.15, while the IQR values for vegetables DSS and fruits DSS were 0.21 and 0.76, respectively. In addition, legumes/lentils DSS and meat/fish/eggs DSS recorded IQR values of 2.56 and 0.74 while milk/ dairy products DSS and total DSS recorded IQR values of 0.45 and 4.96. DSS of all food groups and total were lower than the assigned score, indicating that all food groups and total did not achieve diverse intake status (Table 1). The mean height recorded for all respondents was 130.8 cm, whereas the mean weight was recorded to be 32.1 kg. In addition, the mean BAZ recorded was 0.39, where it was within the normal range of between 1 and 2 (Table 2).

### Diet Diversity and Weight Status of Aboriginal Children

Table 3 shows that among the six food groups, the highest proportion of children to achieve diverse intake status was for the cereal/ grains food group (36.4%), while the highest proportion not to achieve diverse intake status was for the fruits food group (94.2%) which may be influenced by the availability and accessibility of food items respective to food groups (10). Based on the total DSS, 87.9% of the aborigine children did not achieve a diverse diet indicating that children from this study lack the variety of food items intake in their daily diet (Table 2). The differences in the food environment and family characteristics may have an influence on the dietary statuses of children (11).

A total of 60.8% of children were within normal BAZ, whereas 3.4% and 1.4% of them were categorised as thinness and severe thinness. Moreover, 17.7% and 16.7% of children were found to be overweight and obese (Table 2).

### Mean Differences in Dietary Serving Score Across Age Group and Gender of Aborigine

Children aged 7 years old–9 years old significantly consumed more diverse fruits compared to children aged 10 years old–12 years old (29.46 [61.57] versus 18.72 [30.75]; *t* = 1.278; *P* = 0.020). On the other hand, elder aborigine children significantly consumed more diverse meat/fish/eggs (35.68 [41.70] versus 53.42 [62.33]; *t* = −1.870; *P* = 0.029), legumes/ lentils (13.96 [21.00] versus 22.50 [35.12]; *t* = −1.645; *P* = 0.014) and milk/dairy products (1.17 [1.70] versus 2.58 [3.00]; *t* = −3.235; *P* < 0.001) than younger children. However, there were no significant differences in overall total DSS, cereal/grain DSS and vegetable DSS between the age groups of the aborigine children (Table 4). Exposure to a variety of food items during early childhood influences the diversity and quality of diet between younger and elder children (12).

Male children significantly consumed a more diverse intake of vegetables compared to female aborigine school children (3.33 [2.89] versus 2.60 [2.29]; *t* = −1.604; *P* = 0.016) while there were no significant differences between both genders in DSS of other food groups and total (Table 4).

### Mean Differences in BMI-for-Age Across Age Group and Gender of Aborigine Children

There is no significant difference in mean BAZ between age groups of aborigine children (Table 3). In addition, no significant difference in mean BAZ between children’s gender were reported (Table 3). Seventy-four percent and 94.2% of children did not achieve a diverse intake status for vegetable and fruits food groups, respectively, which may be due to the seasonal availability of local fruits (13). Moreover, a lower proportion of children achieving diverse intake status for vegetable food groups because of the liking and preference of the child towards the variety of food items (14).

### Dietary Serving Score and BMI-for-Age Across Sociodemographic Characteristics

Approximately 45.8% of children were within the age group of 7 years old–9 years old, while the remaining was within 10 years old–12 years old. Furthermore, 51.9% were male children. The sub-tribes reported in this study were Temuan (91.5%), Semelai (6.6%) and Jakun (1.9%). A higher proportion of Temuan sub-tribe group reported in study areas was due to the segregation of the sub-tribe aborigine community based on geographic location (Malaysian Administrative Modernisation and Management Planning Unit [MAMPU]) (15). The highest proportion of children was reported for the household of five members (29.9%). Joined families among the aborigine community contributed to larger household size (16). Based on the monthly household income, 89.1% of them reported having a monthly household income below RM2,000. A higher proportion of unemployment status (52.0%) among study samples may have caused a lower monthly household income. Moreover, 78.3% of the study sample reported being the children’s mothers, whereas 19.4% of them were fathers and 2.3% of them were others. In addition, the majority of the parents reported being married (87.8%), 49.3% of the parents completed secondary education, followed by 47.1% and 3.6% of them who completed primary and tertiary education, respectively. As stated previously, more than half of the parents were unemployed, whereas 33.5% of them were self-employed, followed by parents who work in the private sector (8.2%) and government sector (6.3%) (Table 5).

There are no significant mean differences between the DSS score and other socio-demographic characteristics. Although the majority of the aborigine children have a large household size of more than five members and household income level that was categorised under the bottom 40% median household income (B40) category at which the income was below RM4,850 (Department of Statistics Malaysia; DOSM) (17), there is no significant evidence to determine these factors contribute to poor diet diversity among aborigine children in this study (Table 5). Although DSS was reported insignificant statistically with parental marital status, single parents having a lower diverse diet compared to married parents shall not be overlooked (Table 5). The lower diet diversity among single parents is due to financial burden as they are a vulnerable household with low-income socioeconomic status (18). There is a significant difference in mean BAZ across parental employment status (*F* = 3.184; *P* = 0.026), while there were no significant differences across other sociodemographic characteristics (Table 5).

### Relationship between Dietary Diversity and Weight Status

Based on the Spearman correlation test results, cereal/grains DSS (*r* = −0.044; *P* = 0.587), vegetables DSS (*r* = 0.036; *P* = 0.654), fruits DSS (*r* = 0.048; *P* = 0.551), meat/fish/eggs DSS (*r* = 0.086; *P* = 0.290), legumes/lentils DSS (*r* = 0.013; *P* = 0.875), milk/dairy products DSS (*r* = 0.053; *P* = 0.512) and total DSS (*r* = 0.071; *P* = 0.382) with BAZ were reported to be insignificant. Therefore, there is no significant relationship between diet diversity and weight status of aborigine children in this study, indicating dietary diversity status does not determine the increase or decrease of weight status among children. Hence, although the children do not achieve a diverse diet status, it does not influence the increased or decreased of BAZ and may still maintain normal weight status.

Referring to the partial correlation test results, cereal/grains DSS (*r* = −0.170; *P* = 0.063), vegetables DSS (*r* = −0.071; *P* = 0.440), fruits DSS (*r* = 0.009; *P* = 0.918), meat/ fish/eggs DSS (*r* = −0.044; *P* = 0.629), legumes/ lentils DSS (*r* = −0.088; *P* = 0.336), milk/ dairy products (*r* = −0.146; *P* = 0.111) and total DSS (*r* =−0.054; *P* = 0.557) with BAZ were also reported to be insignificant. Hence, there is no relationship between diversity statuses of all food groups and total with weight status of children in this study adjusted to age group, gender and parental employment status (Table 6).

## Discussion

Overall, only cereal/grains, vegetables and milk/dairy products food groups achieved recommended serving size (Table 1). The key findings in this study reported the shared similarity and differences with a study conducted by Chong et al. (19) in which the authors reported that cereal/grains (4.77 ± 0.05), fruits (1.06 ± 0.04), vegetables (1.17 ± 0.07), legumes (0.26 ± 0.01), fish (0.75 ± 0.02) and milk/dairy products (0.59 ± 0.03) food groups failed to achieve the recommended serving size while meat/poultry food group achieved the recommended serving size. However, the authors reported that Malaysian children consumed fruits and vegetables with an average of 0.91 and 1.07, respectively, which indicates that children failed to achieve the recommended serving size.

A study conducted by Ogechi and Chilezie (20) reported that the total mean DDS was 6.04, in which the highest mean scores came from cereal (DDS = 78) and vegetables (DDS = 0.78) food groups while eggs (DDS = 0.15) had the least DDS compared to other food groups (20). Furthermore, the average DDS reported among children in that study was 4.56 out of 9, whereby 91.1% of the study sample was categorised as medium diverse intake category was reported by a study conducted by Sirasa et al. (21). The study also highlighted that fruits (DDS = 1.02) and vegetables (DDS = 0.84) achieved low diverse intake status based on the DDS of respective food groups. To assess diet diversity status, the DSS method is more reliable as it takes into account the number of food items, serving size and age group.

Cordelia et al. (22) reported that 52% of Semai aborigine children were categorised as moderate to severe thinness, which was higher than that reported in this study. Furthermore, Mas-Harithulfadhli-Agus et al. (23) reported that 34.2% and 16.4% were at risk of underweight and thinness, respectively. Nevertheless, a higher proportion of overweight and obesity reported in this study indicates the possibility of severe malnutrition due to poor nutrient-dense food intake and high energy-dense food intake in addition to less diverse food intake (24).

Furthermore, Aurino et al. (25) highlighted on DDS mean score, which shared similarities and contrasting findings with this study. There are also other studies that reported on diet diversity and nutrient adequacy among children (22, 26–27). The authors also reported that boys aged 8 years old significantly consumed a greater overall diverse diet compared to female (DDS = 4.4–4.5 versus 4.2–4.3; *P* = < 0.01) whereas female aged 12 years old significantly consumed a more overall diverse diet (DDS = 4.3–4.4 versus DDS = 4.2–4.3; *P* = <0.01) indicating there might be significant differences between genders on the diet diversity respective to food groups and total. Moreover, Bouhlal et al. (28) highlighted that child’s gender influences parental food choices (Table 4).

Lee et al. (29) reported that elder children tend to have a greater BMI than younger children in addition to the influence of genetic and environmental factors. For instance, elder children may have greater screen time and engage in physical activities less frequently than younger children giving rise to higher BMI and, risk of overweight and obesity. Nevertheless, this study’s lifestyle and environmental effects differ from Lee et al. (29) due to socioeconomic and socio-cultural differences between study samples.

In addition, Pei et al. (30) highlighted financial burden and large household size give rise to food insecurity to access a variety of food items among the aborigine community in Malaysia. Elder children in this study may have a lesser screen time and engage in physical activities such as cycling more often due to limited access to gadgets and other technological appliances. In addition, Kuralneethi et al. (31) reported a similar finding which highlighted that there were no significant differences between BAZ with age and gender of aborigine children in that study. Moreover, the authors also stated that the insignificant differences between both genders were evident as the dietary intakes between both genders were similar.

A similar finding on food diversity intake status was reported by Mahmudiono et al. (32), in which a larger proportion of children (65%) consumed less variety of meat/poultry food groups while 41% of children have a less diverse intake of milk/dairy products. Eggs consumption was reported to be least diversely consumed by 25.2% of children (11). Ogechi and Chilezie (20) reported that the cereal/grains food group (99.2% and 73.5%, respectively) were more diversely consumed among respective study samples compared to other food groups. Moreover, the authors also highlighted a positive association between household income and dietary diversity among study samples. A monotonous diet among the aborigine children may be due to cultural influence as well as food environmental characteristics like availability, accessibility and adequacy (11).

A study by Geik and Sidek (33) reported similar finding, highlighting that there was no significant association between socio-demographic background and weight status. However, height status was significantly associated with household income, household size and gender. Furthermore, Mas-Harithulfadhli-Agus et al. (23) found that there is a significant association between weight status and parental employment status, mother’s education level, and household income level with weight status. In contrast, Amirhamidi et al. (24) reported there is an association between parental education level and employment status with weight status respective to gender. Moreover, the authors also reported that parents with higher household income and job rankings had children with greater BAZ. Pei et al. (30) reported that lower household income gives rise to poorer nutritional status and weight status. Hence, unemployed parents are at higher risk of lower household income than self-employed parents causing more children to have higher BAZ among self-employed parents while children with unemployed parents tend to have a lower BAZ (under-nutrition).

Amirhamidi et al. (24) also reported another contrary findings whereby grains and dairy diversity had a negative association with weight status. Those who reported at the highest quartile of the dietary score of respective food groups were at lower risk of obesity compared to lower and middle quartiles, in addition to the relationship between high energy density and lower diversity intake with increased risk of overweight and obesity among children. Furthermore, there were also other contraries, key findings reported by Hooshmand and Marhamati (34), whereby lower diet diversity has a positive association to weight status among children. The contrast of key findings is caused by several reasons. Firstly, different tools were used to assess the dietary diversity status (DSS method versus DDS method). Secondly, the statistical analysis of this was not adjusted for energy intake. Furthermore, the difference in the number of food groups categorised in each study may influence the key finding.

## Conclusion

In conclusion, diet diversity status has no association with the weight status of aborigine children in this study. Nevertheless, there is still a need for nutrition intervention programmes to improve their dietary diversity and reduce the proportion of children being categorised as overweight and obese.

There are two strengths of this study. Firstly, to the best of our knowledge, this is the first study to determine the dietary diversity status, weight status, and its association among school-aged aborigine children in Malaysia. Moreover, the data and discussion generated from this study can contribute to further studies done in the future by providing information and insights on this field of study.

Nevertheless, this study also has limitations that were recognised by the researchers. To begin, there is a paucity of data due to the limited number of studies done on school-aged aborigine children in Malaysia. In addition, the findings from this study cannot be generalised to Malaysian children of different ethnicities due to socioeconomic and cultural differences. Although the objectives of this study are achieved using DSS method, the quartiles of dietary diversity respective statuses such as lower quartile, middle quartile and higher quartile respective to food groups cannot be assessed among the study sample.

From this study, there are few recommendations to be highlighted for future researches in this field of study. The first recommendation is to have a greater number of sample sizes to represent the aborigine population in Malaysia. Increasing the sample size will also improve the reliability, precision and accuracy of the results of the study. The next recommendation is to include the role of energy and macro and micronutrient intake when studying the association between dietary diversity and weight status, as these may also play a role in the weight status of the study sample. Furthermore, more studies on the diet and nutritional status of school-aged aborigine children need to be done to increase data and insights for upcoming research. Simultaneously, there is also a need to conduct more studies relating to this field of study on Malaysian children to generate results that can be applied in general Malaysian settings.

## Figures and Tables

**Table 1:** Assigned scores for major six food groups based on number of serving

Food group	No. of servings*		Assigned scores

	7–9 (years old)	10–12 (years old)	
Cereal/grains	5	7	4
Vegetables	3	3	4
Fruits	2	2	4
Legumes/lentils	1	1	2
Meat/fish/egg	1	1.5	2
Milk/dairy products	2	2	4

Total diet			20

Notes:

* Recommended serving size based on Malaysian dietary guidelines for children and adolescents; Adapted from Malaysian dietary guidelines for children and adolescents’ website

**Table 2:** Descriptive analysis of SS and DSS of respective to food groups and anthropometry measurements of aborigine children

	Median (IQR)	Mean (SD)	Min.	Max.
SS				
Cereal/grains	4.86 (5.25)		0.00	21.83
Vegetables	1.72 (2.18)		0.00	15.50
Fruits	4.58 (9.54)		0.00	162.26
Legumes/lentils	1.68 (6.41)		0.00	44.00
Meat/fish/eggs	10.86 (18.71)		0.00	123.64
Milk/dairy products	0.45 (1.36)		0.00	7.00
DSS				
Cereal/grains	0.15 (0.15)		0.00	0.70
Vegetables	0.16 (0.21)		0.00	1.48
Fruits	0.37 (0.76)		0.00	12.98
Legumes/lentils	0.67 (2.56)		0.00	17.60
Meat/fish/eggs	0.54 (0.74)		0.00	4.85
Milk/dairy products	0.15 (0.45)		0.00	2.33

Total DSS	2.96 (4.60)		0.00	25.50

Anthropometry measurements				
Height (cm)		130.80 (12.00)	105.10	165.20
Weight (kg)		32.10 (12.30)	16.70	79.70
BAZ		0.39 (1.59)	−4.94	5.170

**Table 3:** Diet diversity status of aboriginal children respective to food groups and total diet

Variables	Frequency (*N*)	Percentage
Cereal/grains diversity (*n* = 173)		
Achieve diverse intake	63	36.4
Did not achieve diverse intake	110	63.6
Vegetables diversity (*n* = 173)		
Achieve diverse intake	45	26.0
Did not achieve diverse intake	128	74.0
Fruits diversity (*n* = 173)		
Achieve diverse intake	10	5.8
Did not achieve diverse intake	163	94.2
Meat/fish/eggs diversity (*n* = 173)		
Achieve diverse intake	38	22.0
Did not achieve diverse intake	135	78.0
Legumes/lentils diversity (*n* = 173)		
Achieve diverse intake	56	32.4
Did not achieve diverse intake	117	67.6
Milk/dairy products diversity (*n* = 173)		
Achieve diverse intake	37	21.4
Did not achieve diverse intake	136	78.6
Total diet diversity (*n* = 173)		
Achieve diverse diet	21	12.1
Did not achieve diverse diet	152	87.9
BAZ (*n* = 293)		
Severe thinness	4	1.4
Thinness	10	3.4
Normal	178	60.8
Overweight	52	17.7
Obese	49	16.7

**Table 4:** Mean difference in DSS and BAZ across age groups and gender

Variables	Age group		*t*-value	*P*-value	Gender		*t*-value	*P*-value
	
	7–9 (years old) *n* = 97 (45.8%)	10–12 (years old) *n* = 115 (54.2%)			Male *n* = 110 (51.9%)	Female *n* = 102 (48.1%)		
	
	Mean (SD)				Mean (SD)			
Cereal/grains	3.62 (2.50)	3.96 (2.45)	−0.719	0.478	3.94 (2.79)	3.64 (2.42)	0.633	0.280
Vegetables	2.72 (2.35)	3.12 (2.79)	−0.870	0.103	2.60 (2.29)	3.33 (2.89)	−1.604	0.016^*^
Fruits	29.46 (61.57)	18.72 (30.75)	1.278	0.020^*^	21.38 (48.29)	26.49 (47.33)	−0.604	0.559
Meat/fish/eggs	35.68 (41.70)	53.42 (62.33)	−1.870	0.029^*^	46.46 (56.73)	43.64 (51.63)	0.294	0.738
Legumes/lentils	13.96 (21.00)	22.50 (35.12)	−1.645	0.014^*^	19.65 (30.95)	17.20 (28.14)	0.467	0.343
Milk/dairy products	1.17 (1.70)	2.58 (3.00)	−3.235	0.000^*^	2.10 (2.59)	1.72 (2.55)	0.845	0.776

Total	86.62 (94.84)	104.30 (108.62)	−0.978	0.385	96.13 (99.59)	96.01 (106.56)	0.006	0.862

BAZ	0.452	1.541	0.198	0.159	0.484	0.343	0.523	0.645

**Table 5:** Mean differences in DSS and BAZ across socio-demographic characteristics

Socio-demographic characteristics	*n*	%	DSS			BAZ		
	
			Mean (SD)	*F*-value	*P*-value	Mean (SD)	*F*-value	*P*-value
Sub-tribe group								
Temuan	194	91.5	5.64 (5.80)	2.46	0.090	0.36 (1.65)	1.291	0.278
Semelai	14	6.6	2.99 (2.97)			1.14 (1.26)		
Jakun	4	1.9	1.37 (1.04)			0.18 (1.89)		
Household size								
Three members and below	17	7.7	4.84 (7.49)	1.329	0.263	0.15 (1.43)	1.695	0.154
Four members	38	17.2	4.33 (4.84)			0.90 (1.78)		
Five members	66	29.9	4.66 (4.73)			0.08 (1.50)		
Six members	37	16.7	7.40 (6.64)			0.77 (2.11)		
Above six members	63	28.5	4.74 (5.30)		0.22 (1.31)			
Monthly household income (RM)								
< 500	67	30.3	6.28 (7.76)	1.670	0.184	0.024 (1.83)	0.604	0.615
500–999	91	41.2	3.19 (3.00)			0.404 (1.68)		
1,000–1,999	39	17.6	2.99 (2.79)			−0.08 (1.47)		
≥ 2,000	24	10.9	5.53 (5.45)			0.78 (1.59)		
Relationship with the children								
Mother	173	78.3	5.21 (5.78)	0.039	0.962	0.48 (1.60)	0.901	0.408
Father	43	19.4	4.92 (4.82)			0.09 (1.76)		
Others	5	2.3	5.53 (0.68)			0.06 (0.66)		
Parental marital status								
Single parent	27	12.2	3.21 (1.69)	3.764^a^	0.055	−0.27 (1.54)	0.197^a^	0.657
Married	194	87.8	5.31 (5.72)			0.46 (1.61)		
Parental education level								
Primary	104	47.1	5.87 (6.44)	0.981	0.378	0.37 (1.68)		
Secondary	109	49.3	4.54 (4.59)			0.39 (1.62)	0.080	0.923
Tertiary	8	3.6	5.47 (6.77)			0.00 (0.51)		
Parental employment								
Government	14	6.3	6.50 (6.01)	1.263	0.290	0.53 (1.52)	3.184	0.026*
Private	18	8.2	2.41 (1.47)			0.31 (1.70)		
Self-employed	74	33.5	5.12 (5.15)			1.03 (1.77)		
Unemployed	115	52.0	5.50 (6.14)			−0.78 (1.63)		

Notes:

a independent *t*-test;

* *P* < 0.05; statistically significant

**Table 6:** Spearman correlation and partial correlation tests between diet diversity and weight status

Variable Weight status	Diet diversity	Spearman correlation		Partial correlation	
		*r*	*P*-value	*r*	*P*-value
BAZ	Cereal/grains DSS	−0.044	0.587	−0.170	0.063
	Vegetables DSS	0.036	0.654	−0.071	0.440
	Fruits DSS	0.048	0.551	0.009	0.918
	Meat/fish/eggs DSS	0.086	0.290	−0.044	0.629
	Legumes/lentils DSS	0.013	0.875	−0.088	0.336
	Milk/dairy products DSS	−0.053	0.512	−0.146	0.111
	Total DSS	0.071	0.382	−0.054	0.557

Notes: 0.00 < *r* < 0.30; *P* > 0.05 indicates insignificance between variables. Partial correlation was adjusted for age, gender and parental occupation as confounding variables
